# Case Report: Combined Cataract Surgery and Minimally Invasive Glaucoma Surgery Provide an Alternative Treatment Approach for Lowe Syndrome

**DOI:** 10.3389/fmed.2022.913229

**Published:** 2022-07-01

**Authors:** Chen Wang, Wenzhe Zhang, Leyi Wang, Wenhui Liu, Hui Guo

**Affiliations:** Department of Ophthalmology, Qilu Hospital, Shandong University, Jinan, China

**Keywords:** congenital cataract, congenital glaucoma, Lowe syndrome, minimally invasive glaucoma surgery, OCRL, case report

## Abstract

We describe the case of a 4-month-old boy who presented with bilateral congenital cataract and high intraocular pressure (IOP) in the left eye, followed by mental retardation and delayed motor development. Genetic investigation revealed the boy had a splicing variant (c.940-11G>A) of the oculocerebrorenal syndrome of Lowe (OCRL) gene. The boy underwent a lensectomy for congenital cataract in his right eye, and lensectomy combined with a 360° suture trabeculotomy to remove the clouded lens and to control IOP of the left eye. During postoperative one-and-a-half-year follow-up, the boy exhibited an improved visual acuity and a well-controlled IOP without the use of topical IOP-lowering medications. Lowe syndrome is a rare multisystemic disorder that is diagnosed through clinical manifestation and genetic testing. The possibility of Lowe syndrome should be considered in patients presenting with typical triad, and genetic analysis should be performed in time to confirm the diagnosis. We recommend combined cataract surgery and minimally invasive glaucoma surgery (MIGS) as a safe, feasible, and efficient method to treat congenital cataract and glaucoma in Lowe syndrome patients.

## Introduction

Lowe syndrome, which was first described in 1952 (1), is a rare X-linked multisystemic disorder characterized by the triad of congenital cataract, intellectual impairment, and proximal renal tubular dysfunction (2). The prevalence of Lowe syndrome is estimated to be approximately 1 in 500,000 in the general population (2). Lowe syndrome is caused by a variant of the OCRL gene, which is located on chromosome Xq25-26 and codes for OCRL-1, an inositol polyphosphate 5-phosphatase (3). OCRL-1 regulates a variety of cellular processes, including cytokinesis, endocytosis, ciliogenesis, phagocytosis, endocytic recycling, and cell migration (4). All Lowe syndrome patients have congenital bilateral cataract during their first ophthalmic examination (5). Cataracts develop *in utero* and are caused by defective development and subsequent degeneration of the primary posterior lens fibers (6). Glaucoma, with or without buphthalmos, is observed in approximately half of Lowe syndrome patients (5, 7).

## Case Description

A 4-month-old boy was brought to our hospital with decreased visual acuity and nystagmus in both eyes, accompanied with photophobia and lacrimation in the left eye for 2 months. He was diagnosed with congenital bilateral cataract and unexplained growth retardation at 3 months old at a local hospital. The boy exhibited a high IOP in his left eye and was admitted to our department for surgical treatment on May 6, 2020. The boy was a full-term infant and weighed 4,050 g at birth. After birth, he was hospitalized at a local hospital for 11 days for neonatal pneumonia and hypoxic-ischemic encephalopathy. He was fed both breast milk and infant formula. There was no contributing medical or familial history, and his parents were in a non-consanguineous marriage.

The boy weighed 6,500 g at the time of admission. During the physical examination, he was slow to respond and insensitive to sound. The boy had a wide eye distance and other facial features including oxycephaly, micrognathia, and facial eczema. His muscle tonus was slightly decreased, and his knee jerk was absent. His head was twisted to the left and his hands could not be centered in the supine position, his head could not be raised in the prone position, and his head was bent back and his body was folded in the sitting position. The boy was diagnosed with bilateral horizontal nystagmus. He was incapable of tracking moving objects or turn his head in the direction of a sound.

The IOP was 20.55 millimeters of mercury (mmHg) in the right eye and 38 mmHg in the left eye using Icare^®^ rebound tonometer (Icare Pro, Finland). The cornea, anterior chamber, and iris were normal in his right eye, but the left eye had a cloudy cornea. The corneal diameter was 11.0 mm in his right eye and 11.9 mm in his left eye at the time of diagnosis. The fundus view was obscured in both eyes due to dense nuclear cataract. The axial length was approximately 19 mm in his right eye and 20 mm in his left eye using B-scan ultrasonography.

Laboratory test revealed that blood routine, blood coagulation function, liver function, and renal function were all within normal limits.

**FIGURE 1:**
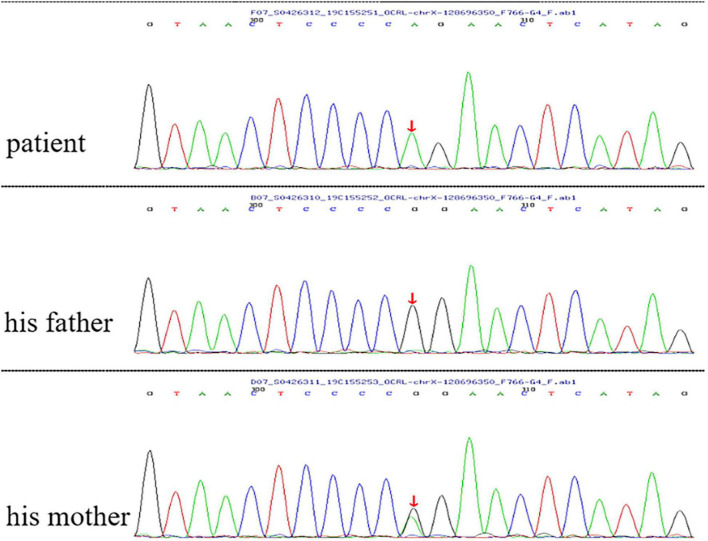
Genetic analysis indicated that the boy had a splicing variant (c.940-11G>A) of the OCRL gene. His mother carried a heterozygous mutation, whereas no mutation was detected in his father. Red arrow indicates the site of the mutant nucleotide.

Genetic testing indicated that the boy had a splicing variant (c.940-11G>A) of the OCRL gene, which had also been reported in a previous case (8). The mutation causes an abnormal splicing and leads to premature termination of the resultant OCRL-1 protein. His mother carried a heterozygous mutation, whereas no mutation was detected in his father (Figure 1). *In silico* and segregation analysis confirmed the pathogenicity of the variant, which was consistent with the diagnosis of Lowe syndrome.

The results of imaging examination such as electroencephalograms and computed tomography of the brain were normal. The bone mineral density revealed severe deficiency, and the speed of sound was 2,730 m per second.

The boy was ultimately diagnosed with Lowe syndrome based on the typical clinical features, ophthalmic examination, and genetic analysis.

## Treatment

**FIGURE 2:**
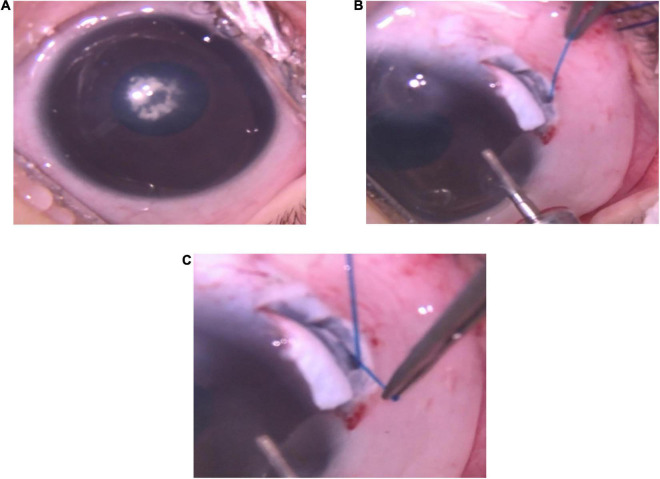
Operative photos of the left eye. **(A)** The cornea was cloudy and nuclear cataract occluded the visual axis. **(B)** The tip of a 5-0 suture was inserted into Schlemm’s canal and threaded circumferentially in Schlemm’s canal during trabeculotomy. **(C)** The tip of the 5-0 suture appeared in the opposite ostium of Schlemm’s canal.

On May 8, 2020, the boy underwent lensectomy, posterior capsulotomy, and anterior vitrectomy in both eyes, as well as a 360° suture trabeculotomy in his left eye. The surgeries were performed under general anesthesia after fully dilatation of pupil. During the cataract operation for the right eye, after central continuous curvilinear capsulorhexis, we found that the lens was not fully developed and manifested cloudy, highly flattened and fragile (Figure 2A). Therefore, we changed the surgical procedure from the preplanned phacoemulsification to carefully remove the lens with a 23-gauge vitrectomy cutter. To maintain the IOP and stabilize the depth of anterior chamber, a drainage needle was inserted into the anterior chamber via the limbal incision. A vitrectomy probe was then inserted into the anterior chamber via the main corneal incision. The Laureate (ALCON, Switzerland) surgical machine was used, with a cutting rate of 250–300 cuts per minute (cpm) and a maximum suction pressure of 450 mmHg. After cataract aspiration, the same vitrector probe was used to perform posterior capsulotomy and anterior vitrectomy to reduce the possibility of postoperative iris synechia formation. The residual anterior and posterior lens capsules were well preserved for secondary intraocular lens implantation.

For the combined surgeries for the left eye, a fornix-based conjunctival flap was dissected and a rectangular 4 × 3 mm size superficial scleral flap with a 1/2 scleral thickness at 12 o’clock was prepared for use. Following the removal of the cataract, posterior capsulotomy and anterior vitrectomy using a 23-gauge vitrectomy cutter as performed in the left eye, a 2 × 2 mm deep scleral flap was dissected to unroof the Schlemm’s canal anterior to the scleral spur. Next, the tip of a 5-0 suture was thermally blunted and inserted into the canal and threaded circumferentially around the canal (Figures 2B,C). The trabecular meshwork was then cleaved circumferentially by pulling both end of the suture. The superficial and deep scleral flaps were then tightly sutured with a 10-0 suture after the viscoelastic agents and hyphema were evacuated.

## Follow-Up and Outcomes

**FIGURE 3:**
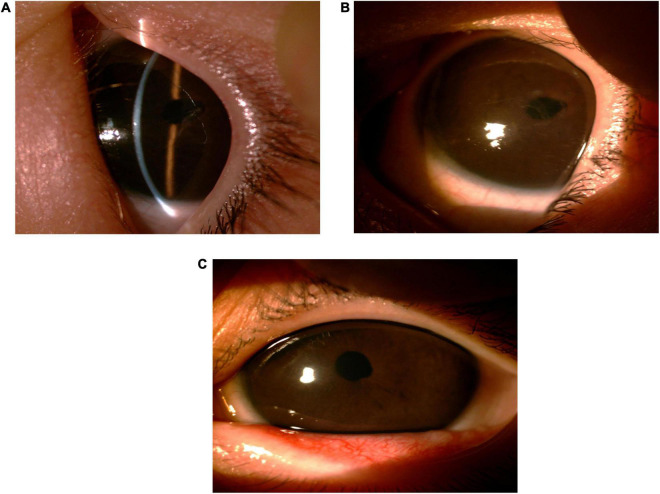
Slit lamp examination of the left eye in routine follow-ups. **(A,B)** Captured 2 months after surgery. **(C)** Captured 14 months after surgery.

On the first postoperative day, an ophthalmic examination revealed a significant reduction in bilateral horizontal nystagmus, and that the boy could track moving objects. The corneas were clear and the anterior chamber depth was normal in both eyes. The diameter of the pupil was 6 mm in the right eye due to subconjunctival injection of 0.2 mg of atropine sulfate after operation and 3 mm in the left eye. Both eyes were aphakic. The steroid eye drops, tobramycin-dexamethasone eye drops (5 mL of tobramycin 0.3% and dexamethasone 0.1%, Alcon, Fort Worth), was applied four times per day for 3 weeks. The tobramycin-dexamethasone eye ointment was applied once a day before sleep for 3 weeks. To prevent posterior synechiae of iris, 1% atropine sulfate ophthalmic gel was applied three times daily to the right eye for 3 months. 1% pilocarpine eye drops were administered three times daily to the left eye for 3 months to prevent peripheral anterior synechiae, which can lead to aqueous humor drainage failure. No postoperative complications, such as posterior synechiae and irregular pupil, occurred even after long-term usage of miotic. Spectacles were used to improve visual function. The IOP was well-controlled without use of medicine in both eyes at one and a half years’ follow-up (Figure 3).

In addition to the ocular manifestations, severe muscular hypotonia and intellectual impairment were typical nervous manifestations in the present case. There was no causative therapy and treatment was symptomatic and supportive. The patient received a regular rehabilitation therapy to treat hypotonia and related complications.

## Discussion

A clinical diagnosis of Lowe syndrome should be considered when a patient presents with bilateral congenital cataract and congenital glaucoma, especially when accompanied by intellectual disability and renal insufficiency. Management includes early lens extraction and prescription of eyeglass, glaucoma treatment and symptomatic and supportive therapy to treat nervous and renal manifestations including muscular hypotonia, intellectual impairment and renal dysfunction.

In general, congenital cataract require surgical intervention to maintain normal visual function. The choice of optimal technique and time for surgery is critical to the effective treatment. In pediatric cataract surgery, a 23-gauge *trans* corneal limbal vitrectomy is recommended as a safer and more effective method than phacoemulsification (9). According to a recent retrospective review, cataract extraction was performed on 132 out of 137 Lowe syndrome patients with a mean age of 4 months and the majority of these patients were aphakic (10). The reason for not recommending intraocular lens implantation are age of the patient during surgery, high prevalence of infantile glaucoma, and poor pupil dilation (5). In this retrospective review, corneal scarring and keloids developed in about 18.2% of patients around the age of 7 years (10). The etiology of corneal keloids remains obscure and it might be related to contact lens wearing and topical antibiotics (11). Therefore, after cataract surgery, eyeglasses are preferred over contact lens to decrease the risk of corneal keloid formation (11). Taking various factors into consideration, cataract surgery should be performed early in the treatment of Lowe syndrome to maximize residual vision stimulation and avoid amblyopia (2).

In this retrospective review, congenital glaucoma occurred in 75 out of 137 Lowe syndrome patients, with a median age of 0.3 years at diagnosis (10). Glaucoma was reported as the main cause of blindness (46%). Over half (55%) of glaucoma patients underwent surgery, whereas the remaining patients used medications to control their eye pressure. The most commonly used drugs were timolol, latanoprost, and dorzolamide/timolol. Different surgical approaches were reported to be used to treat glaucoma, including goniotomy, trabeculotomy, cryotherapy, aqueous tube shunt implantation, and iridectomy. The best outcome was achieved using aqueous tube shunt implantation. Trabeculectomy and goniotomy were the most commonly used treatment methods for glaucoma but with the lowest clinical efficacy score of any of the five aforementioned approaches. However, combined cataract and glaucoma surgeries were not reported in this retrospective review.

Walton et al. indicated that cataract surgery may reduce the success rate of traditional goniotomy due to several negative factors such as lens tissue release and retention in the anterior chamber and steroid administration (7). Minimally invasive glaucoma surgery (MIGS), such as Trab360, gonioscopy-assisted transluminal trabeculotomy (GATT), and Kahook Dual Blade (KDB) has been shown to be safe and effective in treatment in selected cases of childhood glaucoma (12). In the present case, combined lensectomy and a 360° suture trabeculotomy had a significant effect on the treatment of cataract and glaucoma in Lowe syndrome patient, with no related complications during postoperative one-and-a-half-year follow-up. Our result indicated that a 360° suture trabeculotomy may be more effective than traditional goniotomy and lens extraction by lensectomy had no effect upon the success of glaucoma surgery.

Cataract surgery in children tends to be associated with significant inflammation due to vigorous inflammatory response during surgery and healing process. Intensive topical steroids and regular cycloplegic drops were used to reduce the possibility of postoperative inflammatory complications for cataract surgery in the patient’s right eye. However, combined lensectomy and a 360° suture trabeculotomy was performed in his left eye and post-operative pilocarpine was needed to lower the risk of peripheral anterior synechiae after trabeculotomy and improve the chance of better IOP control. Concerning about possible increased incidence of inflammation caused by pilocarpine, an intensive follow-up schedule and meticulous examination for his left eye were arranged to detect possible postoperative complications and modify the treatment strategy timely. Fortunately, no excessive inflammation occurred during the follow-up.

Gene analysis indicated that the boy had a splicing variant (c.940-11G>A) of the OCRL gene, which had been reported in a previous case (8). The mutation predicts premature termination of the OCRL-1 protein. To date, more than 200 different OCRL variants have been described, but no variant is found in 10–20% of patients with suspected Lowe syndrome (13). In families with a known OCRL variant, prenatal diagnosis can be performed by chorionic villi or amniotic fluid sampling (11).

Therefore, it is proposed that combining lensectomy and a 360° suture trabeculotomy may provide a safe, feasible, and efficient treatment option for Lowe syndrome patients with congenital cataract and glaucoma. However, larger sample sizes and longer follow-ups are required in future studies to confirm our findings.

## Conclusion

Lowe syndrome is diagnosed based on clinical manifestations and genetic testing. In the present case, combined lensectomy and a 360° suture trabeculotomy revealed significant efficacy and safety in treating Lowe syndrome patients with congenital cataract and glaucoma. Several advantages of combined surgeries include improved vision, prevention of amblyopia, control of IOP, and reduction in the number of surgeries and general anesthesia. This provides an alternative treatment approach to treat congenital cataract and glaucoma in Lowe syndrome patients.

## Data Availability Statement

The original contributions presented in the study are included in the article/Supplementary Material, further inquiries can be directed to the corresponding author/s.

## Ethics Statement

Written informed consent was obtained from the individual(s), and minor(s)’ legal guardian/next of kin, for the publication of any potentially identifiable images or data included in this article.

## Author Contributions

CW, WZ, LW, and WL: concept and design, and drafting of the manuscript. HG: concept and design and critical revision of the manuscript. All authors commented on previous versions of the manuscript, read and approved the final manuscript, met the International Committee of Medical Journal Editors (ICMJE) criteria for authorship for this article, took responsibility for the integrity of the work as a whole, and have given their approval for this version to be published.

## Conflict of Interest

The authors declare that the research was conducted in the absence of any commercial or financial relationships that could be construed as a potential conflict of interest.

## Publisher’s Note

All claims expressed in this article are solely those of the authors and do not necessarily represent those of their affiliated organizations, or those of the publisher, the editors and the reviewers. Any product that may be evaluated in this article, or claim that may be made by its manufacturer, is not guaranteed or endorsed by the publisher.
