# Supplementary material for: Case Report: Combined Cataract Surgery and Minimally Invasive Glaucoma Surgery Provide an Alternative Treatment Approach for Lowe Syndrome
Source: Front Med (Lausanne). 2022 Jul 1;9:913229. doi: 10.3389/fmed.2022.913229 (PMC9283680; doi:10.3389/fmed.2022.913229)
Supplement: Supplementary file 1 [file Table_1.DOCX]

Supplementary Material

## Supplementary Figures


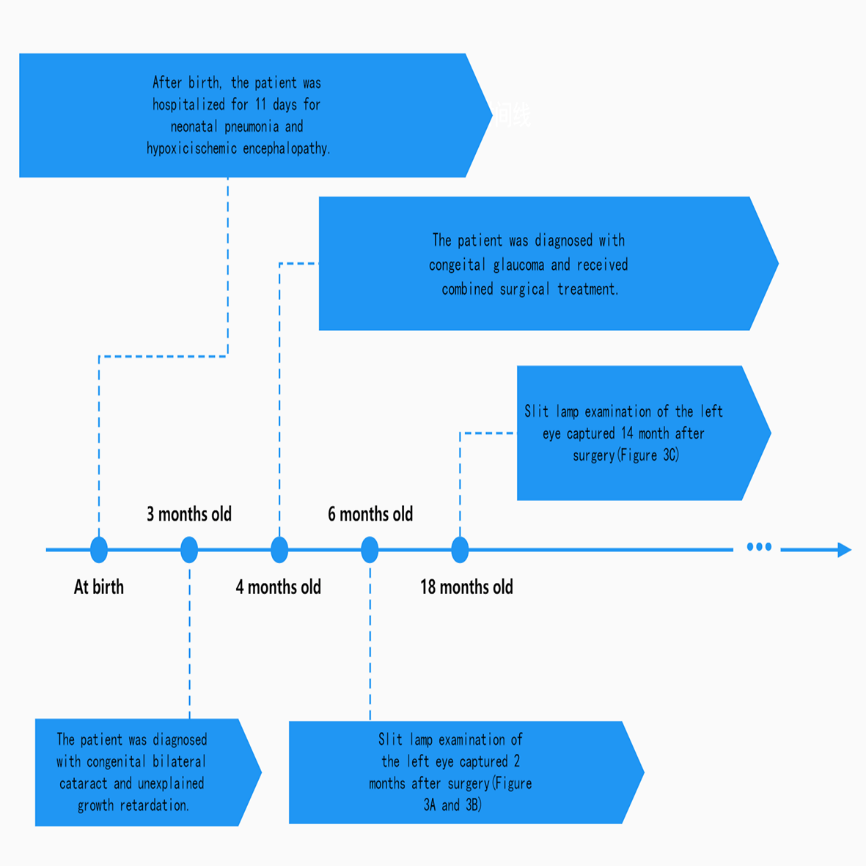


**Supplementary Figure 1.** This is a figure showcasing the case timeline.
